# The sFlt-1/PlGF Ratio at 12, 24, and 32 Weeks Gestation in Twin Pregnancies as a Predictor of Placental Dysfunction

**DOI:** 10.3390/jcm13061784

**Published:** 2024-03-20

**Authors:** Elena Satorres-Pérez, Alicia Martínez-Varea, Blanca Novillo-Del Álamo, José Morales-Roselló, Vicente Diago-Almela

**Affiliations:** 1 Department of Gynecology and Obstetrics, La Fe University and Polytechnic Hospital, 46026 Valencia, Spain; elenasatorres@gmail.com (E.S.-P.); bnalamo@gmail.com (B.N.-D.Á.); cm@comv.es (J.M.-R.); diago_vicalm@gva.es (V.D.-A.); 2 Department of Pediatrics, Obstetrics and Gynecology, Faculty of Medicine, University of Valencia, 46010 Valencia, Spain; 3 Department of Medicine, CEU Cardenal Herrera University, 12006 Castellón de la Plana, Spain

**Keywords:** twin pregnancy, sFlt-1/PlGF ratio, pre-eclampsia, fetal growth restriction, placental dysfunction

## Abstract

**Background:** This study aims to assess the utility of the sFlt-1/PlGF ratio throughout pregnancy in predicting placental dysfunction and neonatal outcomes in twin pregnancies. **Methods:** Prospective study at a tertiary hospital. All pregnant women with a twin pregnancy who signed the informed consent were included. The sFlt-1/PlGF ratio was measured at 12, 24, and 32 weeks’ gestation. **Results:** Seventy patients were included, and 30% developed placental dysfunction. Differences were found in the mean sFlt-1/PlGF ratios at week 32 (13.6 vs. 31.8, *p* = 0.007). Optimal cutoffs at 12, 24, and 32 weeks to identify patients who develop placental dysfunction were 32.5, 8.5, and 30.5, respectively, with ORs of 4.25 (1.13–20.69 95% IC; *p* = 0.044), 13.5 (3.07–67.90 95% IC; *p* = 0.001), 14.29 (3.59–66.84 95% IC; *p* < 0.001). The sFlt-1/PlGF ratio at 32 weeks was associated with gestational age at birth. The sFlt-1/PlGF ratio in weeks 24 and 32 had a statistically significant negative correlation with the birth weight percentile in both twins. **Conclusions:** The potential of the sFlt-1/PlGF ratio as a predictive tool for placental dysfunction in twin pregnancies is underscored.

## 1. Introduction

Complications associated with placental dysfunction, known as placental-related syndromes, are known to have a higher incidence in multiple pregnancies compared to singleton pregnancies [1,2]. The incidence of this condition varies in accordance with risk factors such as assisted reproductive technology, maternal age, parity, or associated comorbidities [3,4,5].

Notably, hypertensive disorders of pregnancy affect 5–6% of singleton pregnancies and 13% of multiple pregnancies [6]. Furthermore, these complications in multiple pregnancies often manifest in more severe forms, demonstrate atypical presentations, and classically manifest earlier than in singleton pregnancies [7]. When the diagnosis is made before 34 weeks, it is defined as early-onset pre-eclampsia (EOPE), compared to later diagnoses, when it is named late-onset pre-eclampsia (LOPE) [8,9]. This distinction must be made, as EOPE carries a heightened risk of life-threatening maternal complications and fetal compromise [10,11].

Fetal growth restriction can be defined as at least one twin with isolated birth weight under the 3rd percentile, or 10th percentile associated with an abnormal Doppler, or, rather, a birth weight discrepancy ≥ 20%, with a reported incidence of 27% and 16%, respectively [12,13]. It is a major cause of stillbirth, preterm birth, admission to intensive care, and poor neonatal outcome [14,15,16], especially in twin pregnancies compared to singletons [17].

In pregnancies characterized by placental dysfunction, specific pro-angiogenic and anti-angiogenic factors present in maternal circulation, namely placental growth factor (PlGF) and soluble fms-like tyrosine kinase 1 (sFlt-1), respectively, undergo alterations, with an altered ratio in favor of anti-angiogenic factors [18,19,20,21,22]. Over the past decade, the sFlt-1/PlGF ratio, serving as an indicator of placental dysfunction and adverse feto-maternal outcomes, has undergone extensive investigation [21,23,24,25]. Its application as a marker for diagnosis, risk stratification, and management in singleton pregnancies is in clinical use, as optimal cutoffs have been established to diagnose and rule out pre-eclampsia with high predictive values [26,27]. Regarding FGR, the only current diagnostic method relies on fetal weight estimation through ultrasound. Although several formulas have been developed to estimate fetal weight [28,29,30,31], their limitations must not be overlooked, as numerous studies reflect a variable accuracy, mostly operator-dependent [32,33]. Developing complementary tools to strengthen sFlt-1/PlGF predictive values could improve the detection of vulnerable cases.

On the contrary, its application in twin pregnancies, in which placental mass, maternal blood volume, and inflammatory response are higher than singletons, is limited [34,35]. Studies evaluating the utility of the sFlt-1/PlGF ratio in twin pregnancies have demonstrated that compared to singleton pregnancies, uncomplicated twin pregnancies have higher sFlt-1/PlGF ratios [36,37,38,39,40]. This is also true in twin pregnancies complicated by pre-eclampsia and FGR [40,41,42]. In addition, in twin pregnancies, higher ratios alone or in combination with other placental biomarkers such as UAPI or PAPP-A can predict [27,36,38,39,41,42] adverse pregnancy outcomes [43,44,45,46,47].

This study aims to assess the ability of sFlt-1/PlGF evaluated at various stages in pregnancy to predict placental dysfunction in twin pregnancies, as well as timing of delivery and neonatal outcomes.

## 2. Materials and Methods

This was a prospective study among mono and dichorionic twin pregnancies that were followed up at the University and Polytechnic Hospital La Fe (Valencia, Spain), from February 2021 to September 2023. The sFlt-1/PlGF ratio was determined at 12, 24, and 32 weeks’ gestation in an outpatient setting through Elecsys^®^ immunoassay of the sFlt-1/PlGF ratio (Roche Diagnostics, Basel, Switzerland). Serum samples were immediately analyzed (<6 h) after the collection. Inclusion criteria were defined: women over 18 years old, pregnant with a twin pregnancy, with no fetal abnormalities, and with signed informed consent. Exclusion criteria included triplets or high-order pregnancies, voluntary abandonment of the study prior to completion, and termination of pregnancy prior to complete sample collection, through birth or stillbirth. Data on patients during pregnancy and delivery were collected from the digital clinical history of the hospital: maternal age, body mass index, parity, toxic habits, chronic diseases, chronic treatments, conception method, chorionicity, amnionicity, weight gain along the pregnancy, diagnosis of gestational diseases (diabetes and hypothyroidism), PAPP-A, UAPI, risk of EOPE calculated in the first trimester, estimated fetal weight in weeks 24 and 32, blood pressure levels at weeks 12, 24, and 32, gestational age at birth, mode and onset of delivery, neonatal outcome (weight, Apgar test, arterial and venous pH, admission to neonatal unit), and puerperal pathology. All these parameters were compared between patients developing placental dysfunction and those who did not. Patients with a previous history of placental dysfunction were not excluded. Registered outcomes were collected through online clinical history and personal interviews if patients gave birth in a different hospital. The primary outcome was defined as placental dysfunction, while secondary outcomes included delivery time and neonatal outcome (birth weight and admission to the neonatal unit). Furthermore, the strength of sFlt-1/PlGF and each outcome correlation, when found significant, was evaluated. All twin pregnancies with the sFlt-1/PlGF ratio measured at 12, 24, and 32 weeks were included in the final analysis. The study group was then split into those who did and did not develop placental dysfunction.

Placental dysfunction was defined as the event of pre-eclampsia, fetal growth restriction, HELLP syndrome, or abruptio placentae [48,49,50].

Pre-eclampsia was defined by the emergence of new-onset hypertension (defined as repeated measurements of systolic blood pressure ≥ 140 mm Hg and diastolic blood pressure ≥ 90 mm Hg) occurring after the 20th week of pregnancy, concomitant with one or both of the following newly arisen conditions: proteinuria (indicated by a urine protein/creatinine ratio ≥ 30 mg/mmol or an albumin/creatinine ratio ≥ 8 mg/mmol or ≥1 g/L [2+] on dipstick testing or 300 mg protein in a 24 h urine collection) or other maternal organ dysfunction, including complications in renal, hepatic, neurological, or hematological domains, or uteroplacental dysfunction (such as fetal growth restriction (FGR), abnormal umbilical artery Doppler waveform analysis, or stillbirth) [37,51,52,53]. The diagnosis of early-onset pre-eclampsia was considered when made prior to 34 weeks, while late-onset pre-eclampsia diagnosis occurred thereafter [11].

HELLP syndrome was characterized by hemolysis (elevated lactate dehydrogenase concentrations surpassing the upper reference threshold), heightened levels of liver enzymes (alanine aminotransferase, aspirate aminotransferase concentrations exceeding the upper reference threshold), and reduced platelet counts (<100,000/μL) [37,51].

Fetal growth restriction (FGR) was established by the combination of an estimated fetal weight (EFW) below the 10th percentile, coupled with either an abnormal Doppler of the umbilical artery (>95th percentile), cerebroplacental ratio (<5th percentile), uterine arteries (>95th percentile), or an EFW below the 3rd percentile [54], in either one or both twins. The EFW was calculated by measurements of head circumference, abdominal circumference, and femoral length, adhering to the formula reported by Hadlock et al. [28]. FGR stages were defined following ISUOG guidelines for twin pregnancies [55].

The 95th percentile of the mean UAPI at 24 weeks was defined as ≥1.35, by prior descriptions [56]. All calculations regarding blood pressure levels were made with the standardized formula (diastolic blood pressure (DBP) + 1/3 [systolic blood pressure (SBP) − DBP]) [57]. Serum PAPP-A hormone levels were determined in the 10th week of pregnancy.

The risk of EOPE was calculated at 11–14 weeks of pregnancy based on the Fetal Medicine Foundation (FMF) algorithm, considering women with values under the 1 in 100 cutoff as high risk [58].

### 2.1. Statistics

The statistical analysis has been carried out using the computer application Rstudio (Version 2023.09.1+494). Quantitative variables were described using standard measures of central tendency and variability: mean, standard deviation, median, interquartile range, maximum, and minimum. Categorical variables were described using frequency and proportion relative to the total number of cases. Bivariate statistical analysis was conducted using a Student’s *t*-test for cases where the normality of the variable was met, and a non-parametric Mann–Whitney test for cases where the assumption of normality was not met. Survival curves were obtained through survival analysis, and diagnostic cutoff curves were determined using ROC curves. Multivariate analysis was performed using linear regression for total gestation days and logistic regression for placental dysfunction.

### 2.2. Ethics

The study was approved by the Ethics Committee of the Health Research Institute Hospital La Fe (IIS La Fe). All pregnant women signed the informed consent form before participating.

## 3. Results

A total of 70 patients with a twin pregnancy were included in our study, both monochorionic (14.29%) and dichorionic (85.71%). In all these collected twin pregnancies, the sFlt-1/PlGF ratio was determined at 12, 24, and 32 weeks of gestation. Data regarding baseline characteristics, comparing pregnant women who did not develop placental dysfunction with those who did, are displayed in Table 1. No significant differences were found between groups.

**Figure 1:**
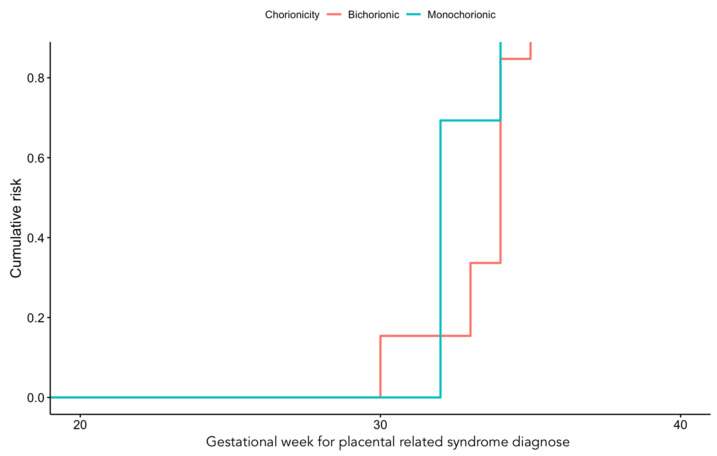
Cumulative risk (weeks) of placental dysfunction in dichorionic and monochorionic pregnancies.

From the final 70 patients included in our sample, 21 developed placental dysfunction (30%). Eleven women suffered from pre-eclampsia, 3 developed early-onset pre-eclampsia, and 6 had late-onset pre-eclampsia (<34 or >34 weeks, respectively). Three of these patients developed severe pre-eclampsia, one of them with associated HELLP syndrome. Furthermore, two patients manifested pre-eclampsia during the postpartum period. Regarding FGR, 10 patients received the diagnosis, 7 of them before 34 weeks of pregnancy. All cases but one suffered from stage I FGR, with the remaining one being diagnosed with stage III FGR and, subsequently, with abruptio placentae. Chorionicity did not statistically impact the gestational age at which placental dysfunction was diagnosed. Figure 1 shows the cumulative risk of placental dysfunction for mono- and dichorionic pregnancies.

**Figure 2:**
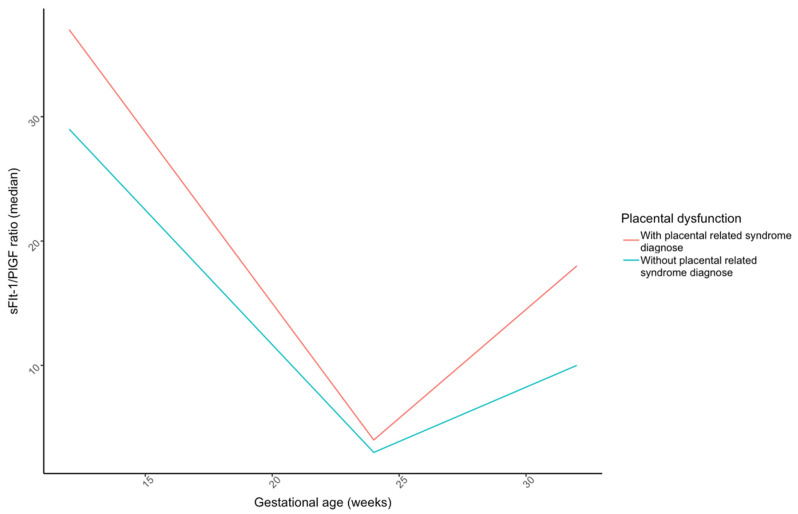
The lines in the figure link the median sFlt-1/PlGF values at each gestational age window for patients diagnosed with placental dysfunction vs. those without. PlGF, placental growth factor; sFlt-1, soluble fms-like tyrosine kinase 1.

The mean maternal serum sFlt-1/PlGF ratio was calculated in three trimesters. As seen in Figure 2 and Table 2, while mean ratios were higher in pregnancies complicated with placental dysfunction (33.0 vs. 46; 4.0 vs. 6.1; 13.6 vs. 31.8, respectively), this difference was only statistically significant at 32 weeks (*p* = 0.007). Furthermore, mean ratio levels were significantly higher in week 32 for women developing early-onset pre-eclampsia compared to late-onset pre-eclampsia (33.9 vs. 12.0; *p* = 0.046).

**Figure 3:**
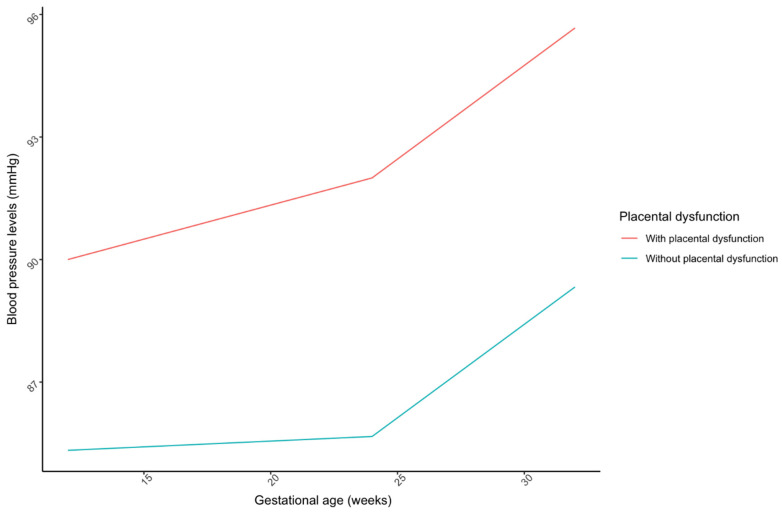
The lines in the figure link the mean blood pressure values at each gestational age window for patients diagnosed with placental dysfunction vs. those without.

Likewise, mean blood pressure levels were considered in the three moments when ratio levels were evaluated. As seen in Figure 3, levels grew throughout pregnancy in both women developing placental dysfunction and healthy patients. Although mean levels were higher in weeks 12, 24, and 32 for patients developing placental dysfunction (85.7 vs. 90.1; 85.7 vs. 88.6; 87.0 vs. 98.2), statistically significant differences were only found in week 32 (*p* < 0.001).

PAPP-A hormone levels at 10 weeks of pregnancy were found to be statistically decreased in twin gestations that developed placental dysfunction compared to those which did not (*p* = 0.044). No statistical differences were found between groups when assessing mean UAPI.

When evaluating gestational age at birth, our sample had a mean value of 36.0 weeks and a median of 36.8 weeks. The mean gestational age at birth was 35.0 for patients with placental dysfunction, compared to 36.4 days without placental dysfunction. Lower ratio levels at week 32 were associated with higher gestational age at birth, as an increase of 1 unit in the ratio at week 32 implied 0.278 days fewer of pregnancy (*p* < 0.005). Table 3 summarizes neonatal outcomes.

**Figure 4:**
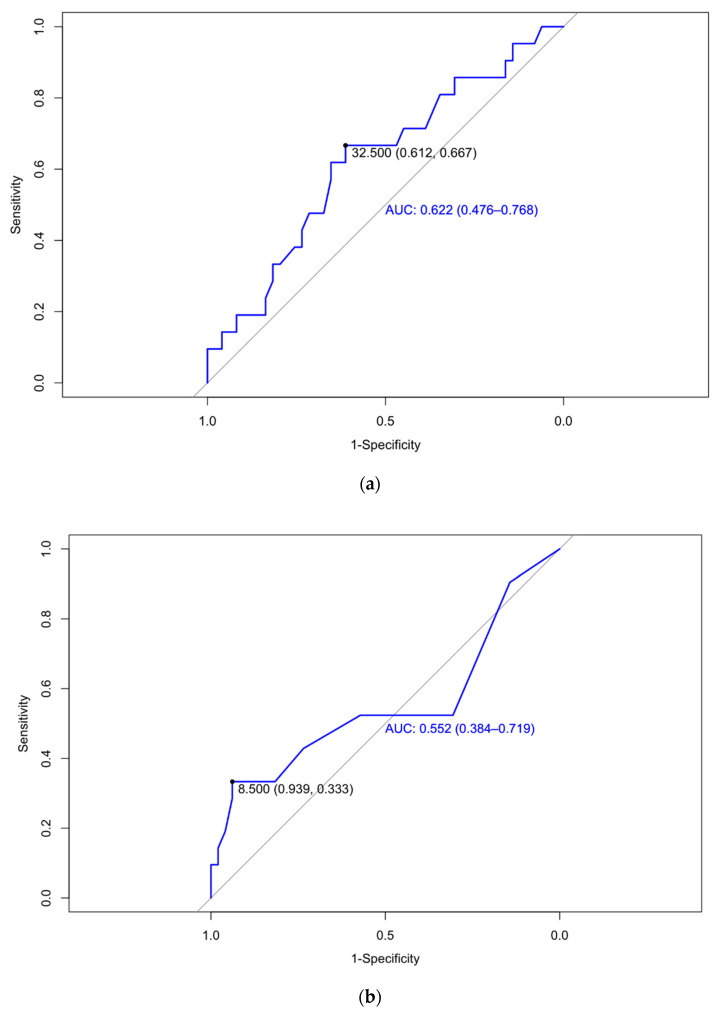

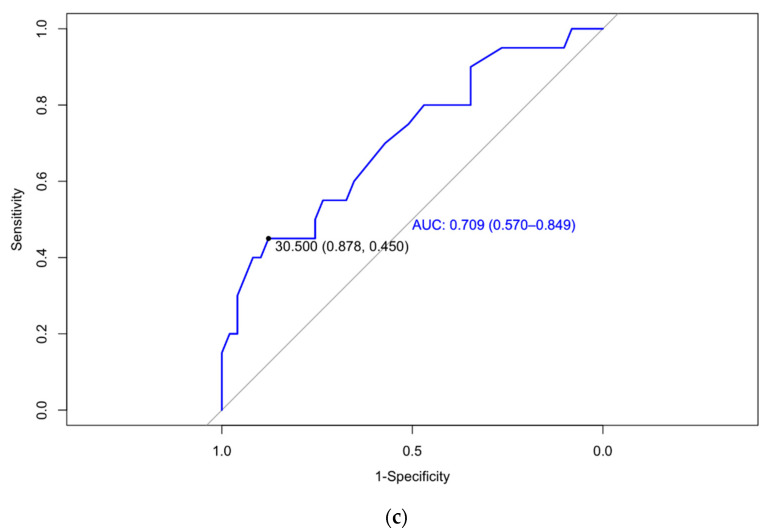
The area under the curve of the sFlt-1/PlGF ratio at 12 (**a**), 24 (**b**), and 32 weeks’ (**c**) gestation for the detection of patients with twin pregnancies who subsequently develop placental dysfunction.

In order to calculate the best cutoff for placental dysfunction prediction, AUCs were designed for each determination. As seen in Figure 4, a cutoff sFlt-1/PlGF ratio ≥ 32.5 at 12 weeks of pregnancy was associated with a sensitivity of 66.7%, a specificity of 61.2%, a PPV of 42.4%, and an NPV of 81.1% for the identification of patients with a twin pregnancy who developed placental dysfunction, with an AUC of 0.622 (0.476–0.768). The OR was 4.25 (1.13–20.69 95% IC; *p* = 0.044). Equally, a cutoff sFlt-1/PlGF ratio ≥ 8.5 at 24 weeks was associated with a sensitivity of 33.3% and a specificity of 93.9%, a PPV of 70%, and an NPV of 76.7% for the identification of patients with a twin pregnancy who develop placental dysfunction, with an AUC 0.552 (0.384–0.719) and an OR of 13.5 (3.07–67.90 95% IC; *p* = 0.001). Furthermore, cutoff sFlt-1/PlGF ratio ≥ 30.5 at 32 weeks was related with a sensitivity of 45%, a specificity of 87.8%, a PPV of 60%, and an NPV of 79.6% for the identification of patients who developed placental dysfunction, with an AUC 0.709 (0.570–0.849) and an OR of 14.29 (3.59–66.84 95% IC; *p* < 0.001).

There was a significant and strong association between the week in which pre-eclampsia was diagnosed and the ratio level in week 12 (Pearson correlation coefficient [PCC] −0.81; *p* < 0.05). This association was negative, meaning that an increase in the values of the ratio at week 12 implied an earlier development of pre-eclampsia. This association was maintained when assessing PlGF levels only, but with a moderate-to-high and positive correlation: an increase in the values of PlGF in week 12 implied a later development of the disease (PCC 0.77; *p* < 0.05). The sFLT-1 isolated value showed an almost significant increase in the diagnosis week for FGR only (PCC −0.38; *p* < 0.10).

**Figure 5:**
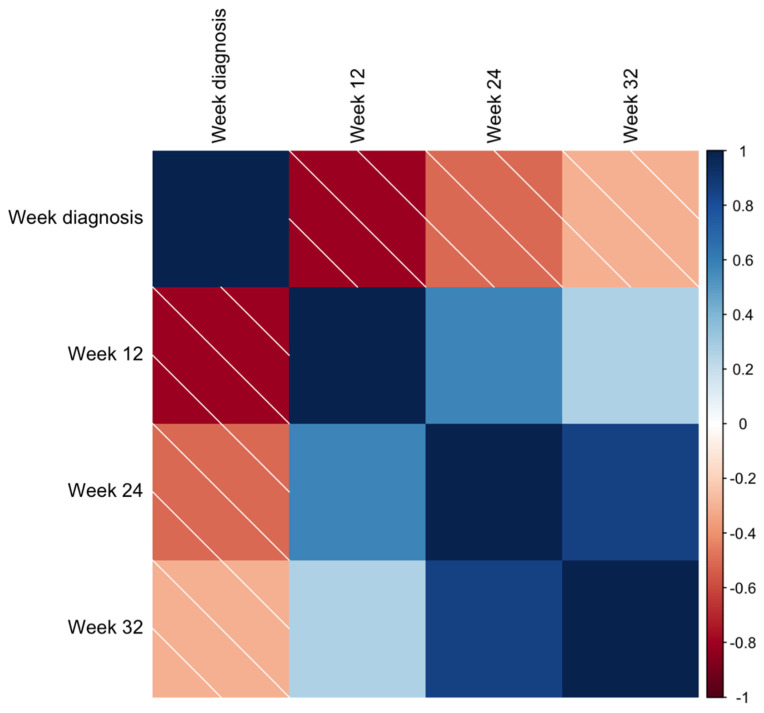
Representation of correlations between sFlt-1/PlGF ratio at 12, 24, and 32 weeks and the week of pre-eclampsia diagnosis. The intensity of the correlation between such variables is represented by the intensity of the color code, as explained in the legend.

Similarly, when assessing ratio levels in week 24 and the time of placental dysfunction diagnosis, differences were only found between the week of FGR diagnosis and the PlGF value. The intensity of this relationship was not very high and positive, meaning that a decrease in PlGF values implied a decrease in the week of diagnosis (0.43; *p* < 0.05). These associations are represented in Figure 5.

No correlation was found between mean blood pressure levels in the three trimesters or mean UAPI levels in the first trimester with the week of placental dysfunction diagnosis.

Regarding birth weight percentile, ratio levels at weeks 24 and 32 had a statistically significant negative correlation, meaning a higher ratio implied a lower birth weight in both newborns (PCC −0.29 and −0.36; −0.41 and −0.33, *p* < 0.005, respectively).

**Figure 6:**
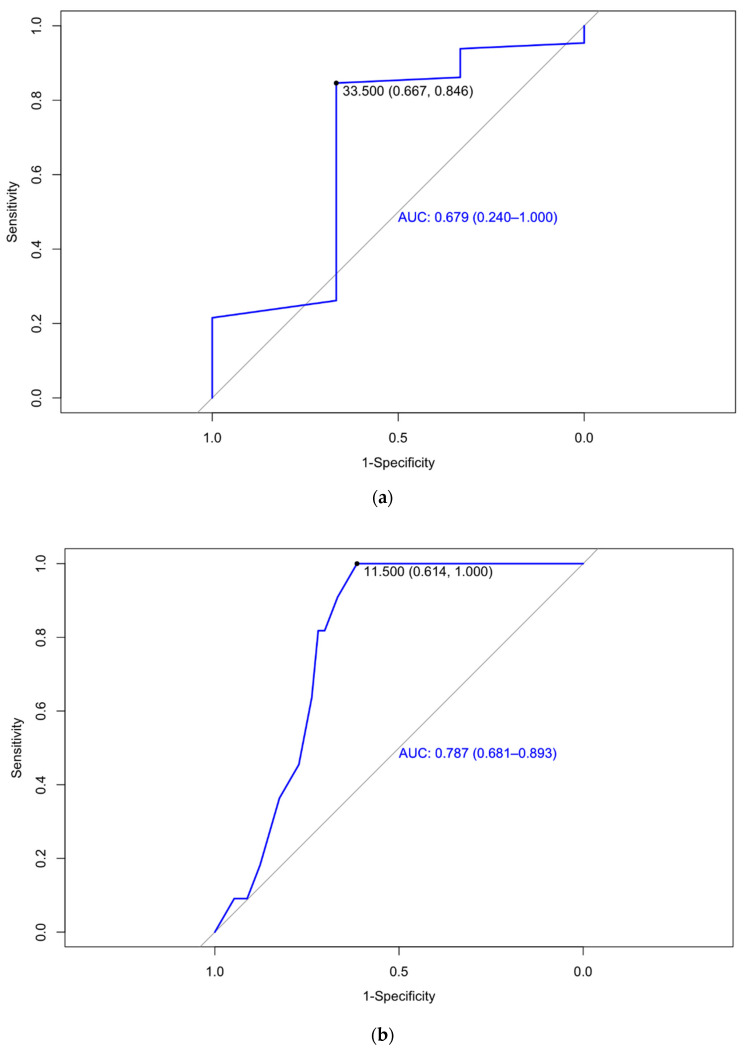
The areas under the curve of the sFlt-1/PlGF ratio at 24 (**a**) and 32 weeks’ gestation (**b**) for the detection of newborns from twin pregnancies with birth weights under 1500 g and 2500 g.

As seen in Figure 6, an sFlt-1/PlGF ratio over 33.5 at 24 weeks had a sensitivity of 66.7% and specificity of 84.6%, with a PPV of 98.21% and NPV of 16.67%, and an AUC of 0.679 (0.240–1.00) to predict a weight under 1500 g, with an OR of 0.09 (0.00–1.03 95% IC; *p* = 0.059). Moreover, an sFlt-1/PlGF ratio over 11.5 at 32 weeks had a sensitivity of 61.4% and specificity of 100% with a PPV of 33.33, an NPV of 100%, and an AUC of 0.787 (0.681–0.893) to predict a weight under 2500 g in one or both newborns.

As for the days of admission to the neonatal unit, there was a significant and positive association with ratio levels at 12, 24, and 32 weeks (PCC 0.36, 0.50, and 0.62, respectively). Hence, the ratio level appears to have an increasing capacity to predict neonatal unit admission throughout pregnancy. Additionally, the median value of the sFlt-1/PlGF ratio at week 12 was 36.5 in the group with admission required compared to 27.5 in the group without admission to the neonatal unit, with an almost significant statistical difference (*p* < 0.10). Noticeably, the ratios at weeks 24 and 32 had median values of 3.0 and 10.0 among cases without admission versus 4.0 and 23.5 among those who did, respectively, in both cases, with significant differences (*p* < 0.05).

Table 4 and Table 5 summarize the sFlt-1/PlGF ratio’s performance for placental dysfunction and neonatal outcome.

## 4. Discussion

The present study reveals the potential predictive value of the sFlt-1/PlGF ratio in twin pregnancies for predicting pre-eclampsia and fetal growth restriction, preterm delivery, and adverse perinatal outcomes. In our sample, sFlt-1/PlGF ratio levels were higher during the three trimesters in twin pregnancies which develop placental dysfunction compared with those who do not, with statistical differences found at week 32.

An sFlt-1/PlGF cutoff ratio ≥ 32.5 at 12 weeks was associated with a significant increase in the frequency of placental dysfunction (odds ratio [OR], 4.25 [1.13–20.69 95% IC]; *p* = 0.044). Additionally, the present study reveals that such a cutoff at 12 weeks is associated with a sensitivity of 66.7%, a specificity of 61.2%, a PPV of 42.4%, and an NPV of 81.1% to detect placental dysfunction (Table 4). These figures surpass the effectiveness of the traditional method for identifying high-risk patients for pre-eclampsia based on maternal demographic characteristics and medical history, as this approach can only identify approximately 40% of preterm cases with a false positive rate of 10% [59,60]. Nevertheless, currently, most centers use the Fetal Medicine Foundation algorithm to evaluate the risk of pre-eclampsia throughout the pregnancy, integrating information on various risk factors, including placental perfusion (assessed through UAPI and mean arterial pressure), clinical characteristics (maternal factors and medical history), and biomarker levels (PlGF). This combined screening has been shown to detect 75% of preterm pre-eclampsia (<37 weeks) and 41% of term pre-eclampsia (≥37 weeks) with a 10% false positive rate [61]. Nevertheless, these detection rates have been calculated based on single pregnancies. An investigation published in 2020 evaluated the best performance of first-trimester screening for pre-eclampsia in twin pregnancies by collecting 3938 twin pregnancies with 339 (8.6%) cases of pre-eclampsia. The best results were achieved by combining maternal factors, MAP, UtA-PI, and PlGF, with a sensitivity of 86.4% and 41.1% for PE at <32 weeks and <37 weeks, respectively, vs. 30.6% and 24.9% by maternal factors alone, with a 10% false positive ratio [62]. Another study showed an AUC of 0.647 (0.604–0.690; 95% CI) for detecting pre-eclampsia in twin pregnancies by combining maternal demographic characteristics and medical history [63], which is very similar to our AUC of 0.622 (0.476–0.768) with only the sFlt-1/PlGF ratio.

In singleton pregnancies characterized by a heightened risk of pre-eclampsia or fetal growth restriction (FGR), as defined by maternal history and second-trimester uterine artery Doppler assessment, the sFlt-1/PlGF ratio at 24–28 weeks has emerged as a precise prognostic indicator for the occurrence of pre-eclampsia or FGR with an area under the curve (AUC) of 0.98 (0.97–1.00; 95% CI) [64]. Individuals with twin pregnancies have revealed a twofold increased likelihood of developing pre-eclampsia when compared to women with singleton pregnancies [1,2,65]. In our study, a cutoff sFlt-1/PlGF ratio ≥ 8.5 at 24 weeks showed a significant increase in the frequency of placental dysfunction (OR, 13.5 [3.07–67.90; 95% CI]; *p* = 0.001), just as for week 32, where a cutoff sFlt-1/PlGF ratio ≥ 30.5 had an even higher rise in both pre-eclampsia and FGR (OR, 14.29 [3.59–66.84 95% CI]; *p* < 0.001). Furthermore, this cutoff at 24 weeks is associated with a sensitivity of 33.3% and a specificity of 93.4%, a PPV of 70%, and an NPV of 76.7%. This finding is in line with our recently published studies, as an sFlt-1/PlGF ratio ≥ 17 at 24 weeks in twin pregnancies has been described as associated with a significant increase in the frequency of pre-eclampsia (OR, 37.13 [4.78–288.25; 95% CI]; *p* = 0.002) and FGR (OR, 39.58 [6.31–248.17; 95% CI]; *p* < 0.001) [40], and the 5th percentile, median, and 95th percentile of the sFlt-1/PlGF ratio in uneventful twin pregnancies at 24 plus 0–28 plus 6 weeks’ gestation has been labeled 1.33, 3.88, and 19.0, respectively [66]. For week 32, a ratio determination ≥ 30.5 was associated with a sensitivity of 45%, a specificity of 87.8%, a PPV of 60%, and an NPV of 79.6% for identifying patients with a twin pregnancy who develop pre-eclampsia or FGR. Consequently, these findings suggest that the successive assessment of the sFlt-1/PlGF ratio holds the potential to increase the predictive accuracy for placental dysfunction diagnoses in twin pregnancies.

Furthermore, the sFlt-1/PlGF ratio has demonstrated a markedly elevated level in singleton pregnancies developing early-onset pre-eclampsia or fetal growth restriction (FGR) when compared to those manifesting a later onset [67]. In the current study, the average sFlt-1/PlGF ratio at 32 weeks of gestation was equally observed to be higher in individuals with twin pregnancies who subsequently experienced the development of early-onset pre-eclampsia or FGR, in contrast to those who encountered late-onset pre-eclampsia or FGR (33.9 vs. 12.0; *p* = 0.046). These results are in line with the observations made by S Rana et al., who found sFlt-1/PlGF ratio levels were higher among women presenting pre-eclampsia prior to 34 weeks compared to those manifesting a later onset (97.7 [76.6–178.1] vs. 31.7 [6.5–48.7]; *p* = 0.001) [42]. Therefore, patients at risk of developing these diseases in an early stage of pregnancy could especially benefit from a close follow-up.

Isolated PlGF levels have a proven capability of predicting pre-eclampsia and preterm delivery for single pregnancies, and fetal growth-restricted pregnancies have been described as exhibiting diminished PlGF levels across the entire pregnancy, especially during the first trimester [20,24,68]. When assessing sFlt-1 and PlGF alone in our sample, statistically significant differences were found between the week of FGR diagnosis and PlGF value in weeks 12 and 24. This is in accordance with a prospective, multicenter observational study published in 2018, where measurement of PlGF in singleton pregnancies was shown to be a valuable adjunct for identifying those at high risk of delivering an SGA infant (with a sensitivity of 93% [95% CI, 84–98%] and an NPV of 90% [76–97; 95% CI), allowing appropriate surveillance and timely intervention [69]. Other studies, including a prospective series published by our group, have similar results, with higher sFlt-1/PlGF ratios [40,42], and lower isolated PlGF and higher sFlt-1 levels in twin pregnancies developing FGR [42]. Consequently, understanding the maternal levels of PlGF may aid in anticipating pregnancies at risk of fetal growth restriction (FGR) complications.

A positive correlation between the sFlt-1/PlGF ratio and the likelihood of pregnancy complications, such as preterm delivery, has been described [70,71,72]. In our study, low levels of the sFlt-1/PlGF ratio were associated with higher gestational age at birth (an increase of 1 unit in the ratio implied 0.278 days fewer of pregnancy [*p* < 0.005]). Noticeably, our study has also shown that ratio levels at weeks 24 and 32 show a statistically significant negative correlation with birth weight percentile, as an sFlt-1/PlGF ratio over 33.5 at week 24 had a sensitivity of 66.7% and specificity of 84.6% to predict a weight under 1500 g. An sFlt-1/PlGF ratio over 11.5 had a sensitivity of 61.4% and specificity of 100% to predict a weight under 2500 g. Regarding the requirement of neonatal admission in a specific unit, differences were found in mean ratio levels at weeks 24 and 32 (3.0 vs. 4.0 and 10.0 vs. 23.5, respectively). Plus, a significant, positive, and progressive association between the ratio levels in the three trimesters and the number of days of admission was determined (PCCs 0.36, 0.50, and 0.62, respectively).

Concerning chorionicity, some previous studies have defended the assertion that sFlt-1/PlGF ratio levels are independent of this variable, corroborating its predictive accuracy in both groups [38,40,41,66]. Nevertheless, another study did find maternal sFlt-1/PlGF ratio higher in monochorionic than in dichorionic patients after adjustment for gestational age [73]. In our sample, no statistical differences were found regarding placental dysfunction incidence between monochorionic and dichorionic pregnancies (*p* > 0.05), nor in sFlt-1/PlGF ratio levels in weeks 12, 24, and 32 (*p* = 0.154; 0.633; 0.828, respectively). This was probably due to the relatively low number of twin pregnancies included, especially monochorionic ones.

## 5. Strengths and Limitations

The main limitation of the present study resides in its constrained sample size, a factor that influenced several variables such that they demonstrate a discernible trend without achieving statistical significance. The limited statistical power stemming from the sample size constrains the ability to draw definitive conclusions and underscores the need for caution in generalizing the findings. Future research with larger cohorts is imperative to corroborate and strengthen the observed trends, thereby enhancing the overall robustness and validity of the study outcomes.

On the other hand, the current study also delineates sFlt-1/PlGF ratio levels in healthy twin pregnancies and those developing placental dysfunction. It proves the sFlt-1/PlGF ratio correlates with gestational age at delivery, birth weight, neonatal outcome, and neonatal admission in an intensive care unit. These associations underscore the potential impact of monitoring the sFlt-1/PlGF ratio in twin pregnancies, as it could provide clinicians with valuable information for managing a condition with a considerable impact on perinatal and neonatal health.

## 6. Conclusions

In this study, the sFlt-1/PlGF ratio in twin pregnancies emerges as a potential predictive tool for adverse feto-maternal outcomes, including pre-eclampsia, fetal growth restriction, preterm delivery, and perinatal outcome. The analysis reveals that an sFlt-1/PlGF ratio ≥ 32.5 in the first trimester is associated with a significant increase in the frequency of placental dysfunction. The predictive capacity of this tool at 12 weeks, compared to traditional methods based on maternal demographic characteristics and medical history, shows a superior sensitivity (66.7%), specificity (61.2%), PPV (42.4%), and NPV (81.1%) for detecting placental dysfunction. Our findings also highlight the potential benefits of sequential assessments of the sFlt-1/PlGF ratio in enhancing predictive accuracy, as cutoffs of ≥8.5 at 24 weeks and ≥30.5 at 32 weeks are associated with increased incidence of both pre-eclampsia and FGR in twin pregnancies. Furthermore, lower ratios at 12, 24, and 32 weeks of pregnancy are associated with other neonatal outcomes such as higher gestational age at birth, higher birth weight percentiles, and the need for admission to a neonatal intensive care unit. This investigation provides valuable evidence regarding the utility of the sFlt-1/PlGF ratios at 12, 24, and 32 weeks as a predictive tool for placental dysfunction and neonatal outcomes in twin pregnancies.

## Figures and Tables

**Table 1:** Description of maternal baseline characteristics of the included patients with twin pregnancies, comparing women who did not develop placental dysfunction with those who did. Data are given as mean (standard deviation) for continuous variables and n (%) for categorical variables. Statistical analysis was made comparing cases and controls. BMI, body max index; ART, artificial reproduction techniques; PE, pre-eclampsia.

	Twin Pregnancies without Placental Dysfunction (n = 49)	Twin Pregnancies with Placental Dysfunction (n = 21)	*p* Value
BMI	23.7 (4.0)	24.1 (4.3)	0.776
Age	35.0 (4.6)	35.8 (4.5)	0.512
ART	26 (53.06%)	14 (66.67%)	0.172
Nulliparous	35 (71.42%)	17 (80.95%)	
Smoking	6 (12.24%)	1 (4.76%)	
Ethnicity: Caucasian	46 (93.88%)	20 (95.24%)	
Ethnicity: Black	1 (2.04%)		
Ethnicity: Asian	2 (4.08%)	1 (4.76%)	
Dichorionic	44 (89.80%)	16 (76.19%)	0.154
Monochorionic	5 (10.20%)	5 (23.81%)	
High-risk of early-onset PE in first trimester	5 (10.20%)	4 (19.05%)	
Low-risk of early-onset PE in first trimester	43 (87.76%)	16 (76.19%)	
PAPP-A levels, week 10	6.6 (9.2)	3.4 (2.6)	0.044
Mean uterine artery pulsatility index	1.2 (0.4)	1.4 (0.5)	0.134
Blood pressure, week 12	85.7 (9.3)	90.1 (11.2)	0.069
Blood pressure, week 24	85.7 (8.4)	88.6 (10.9)	0.238
Blood pressure, week 32	87.0 (9.2)	98.2 (10.9)	<0.001

**Table 2:** Mean values (interquartile range) for the sFlt-1/PlGF ratio and sFlt-1 and PlGF immunoassay values in women with twin pregnancies, with and without placental dysfunction, in the three trimesters of pregnancy.

	Twin Pregnancies without Placental Dysfunction (n = 49)	Twin Pregnancies with Placental Dysfunction (n = 21)	*p* Value
sFlt-1/PlGF ratio—week 12	33.0 (19.2)	46.0 (36.0)	0.109
sFlt-1/PlGF ratio—week 24	4.0 (2.9)	6.1 (5.7)	0.496
sFlt-1/PlGF ratio—week 32	13.6 (12.0)	31.6 (32.8)	0.007
sFlt-1—week 12	2541.4 (918)	2350.3 (902.7)	0.19
sFlt-1—week 24	3258.5 (1585)	3513.8 (1636.5)	0.31
sFlt-1—week 32	5917.4 (4318)	6975.7 (4644.5)	0.14
PlGF—week 12	106.2 (73.3)	69.4 (34.1)	0.021
PlGF—week 24	1184.7 (577)	854.4 (702.7)	0.11
PlGF—week 32	852.6 (619)	386.6 (343.7)	0.02

**Table 3:** Description of neonatal characteristics, comparing those born after placental dysfunction with those without the diagnosis. Data are given as mean (standard deviation) for continuous variables.

	Twin Pregnancies without Placental Dysfunction (n = 49)	Twin Pregnancies with Placental Dysfunction (n = 21)	*p* Value
Gestational age at birth (days)	254.9 (8.4)	245.2 (16.2)	0.004
Weight first newborn	2451.7 (420)	2072 (450.7)	0.002
Weight second newborn	2373.7 (444.8)	2015.2 (507.1)	0.007
Apgar 5′ for first newborn	9.9	9.6	0.389
Apgar 5′ for second newborn	9.8	9.6	0.256
Arterial pH first newborn	7.3 (0.1)	7.3 (0.1)	0.317
Arterial pH second newborn	7.3 (0.1)	7.3 (0.1)	0.989
Admission days	2.5 (6.4)	13.4 (21.3)	0.001

**Table 4:** sFlt-1/PlGF ratio cutoff performance in each gestational week for placental dysfunction and birth weight prediction.

	Prediction	Cutoff	AUC	Sensitivity	Specificity	PPV	NPV
Week 12	Placental dysfunction	32.5	0.622 (0.476–0.768)	66.7	61.2	42.4	81.1
Week 24	Placental dysfunction	8.5	0.552 (0.384–0.719)	33.33	93.88	70.00	76.67
	Birth weight under 1500 g	33.5	0.679 (0.240–1.00)	66.7	84.6	98.21	16.67
Week 32	Placental dysfunction	30.5	0.709 (0.570–0.849)	45	87.8	60	79.6
	Birth weight under 2500 g	11.5	61.4	100	33.33	100	0.787 (0.681–0.893)

**Table 5:** Statistically significant correlations of isolated sFlt-1, PlGF, and sFlt-1/PlGF and studied event (week of pre-eclampsia and fetal growth restriction [FGR] diagnosis, days of neonatal admission and birth weight) determined by Pearson correlation coefficient (PCC).

	Measurement	Event	PCC
Week 12	sFlt-1/PlGF ratio	Week of pre-eclampsia diagnosis	−0.81; *p* < 0.05
		Days of neonatal admission	0.36; *p* < 0.05
	PlGF	Week of pre-eclampsia diagnosis	0.77; *p* < 0.05
	sFlt-1	Week of pre-eclampsia diagnosis	−0.38; *p* < 0.10
Week 24	sFlt-1/PlGF ratio	Birth weight (both newborns)	−0.29 and −0.36; *p* < 0.05
		Days of neonatal admission	0.50; *p* < 0.05
	PlGF	Week of FGR diagnosis	0.43; *p* < 0.05
Week 32	sFlt-1/PlGF ratio	Birth weight (both newborns)	0.41 and −0.33; *p* < 0.05
		Days of neonatal admission	0.62; *p* < 0.05

## Data Availability

The data in this study were obtained from the clinical program of La Fe University and Polytechnic Hospital. Further inquiries can be directed to the corresponding author.
